# Anticholinergic drug exposure is associated with delirium and postdischarge institutionalization in acutely ill hospitalized older patients

**DOI:** 10.1002/prp2.310

**Published:** 2017-05-11

**Authors:** Angelique Egberts, Saskia T. van der Craats, Melissa D. van Wijk, Shams Alkilabe, Patricia M. L. A. van den Bemt, Francesco U. S. Mattace‐Raso

**Affiliations:** ^1^ Section of Geriatric MedicineDepartment of Internal MedicineErasmus University Medical CenterRotterdamThe Netherlands; ^2^ Department of Hospital PharmacyErasmus University Medical CenterRotterdamThe Netherlands

**Keywords:** Adverse effects, anticholinergic drug scoring systems, delirium, outcomes, physical function, prognosis

## Abstract

Several studies investigated the possible association between anticholinergic drugs and diverse clinical outcomes in older persons, but the results are inconsistent. The aim of this study was to investigate whether anticholinergic drug exposure is associated with delirium on admission, length of hospital stay, postdischarge institutionalization and in‐hospital mortality in acutely ill hospitalized older patients. In this observational chart review study, we included acutely ill patients aged 65 and older who were admitted to the geriatric ward of the Erasmus University Medical Center, Rotterdam, The Netherlands, between 2012 and 2015 (*n* = 905). Anticholinergic drug exposure on admission was defined as the use of anticholinergic drugs, total number of anticholinergic drugs and anticholinergic drug burden score (ADB), quantified with the Anticholinergic Risk Scale (ARS), the Anticholinergic Cognitive Burden scale (ACB) and the list of Chew et al. (Chew). Logistic regression analyses were performed to investigate possible associations between anticholinergic drug exposure and the aforementioned outcomes. Analyses were adjusted for age, sex, comorbidities, non‐anticholinergic drugs and delirium, where appropriate. Moderate and high ADB measured with the ARS were associated with delirium on admission with odds ratios (OR) of 1.70 (95% confidence interval (CI) = 1.16–2.49) and 1.83 (95% CI = 1.06–3.15), respectively. High ADB measured with the ARS was also associated with postdischarge institutionalization (OR = 2.43, 95% CI = 1.24–4.75). No associations were found using the ACB and Chew. Future studies are warranted to investigate the clinical usefulness of the ARS in reducing complications in older persons.

**Abbreviations**
ACB: anticholinergic cognitive burden scale
ADB: anticholinergic drug burden
ARS: anticholinergic risk scale
CCI: charlson comorbidity index
Chew: list of Chew et al.
eGFR: estimated glomerular filtration rate
GEE: generalized estimating equations
LOS: length of hospital stay

## Introduction

Drugs with anticholinergic properties are commonly prescribed in older persons (Collamati et al. 2016). These drugs are associated with a wide spectrum of adverse effects including dizziness, blurred vision, urinary retention, constipation, confusion and possibly also delirium (Collamati et al. 2016). Older persons are more susceptible to those adverse effects due to an age‐related increase in blood‐brain barrier permeability, a reduction in hepatic and renal clearance and a decrease in cholinergic neurons and receptors (Collamati et al. 2016; Zeevi et al. 2010; Mangoni and Jackson 2004).

It has been hypothesized that adverse effects of anticholinergic drugs can restrict older persons in performing daily activities and lead to hospitalizations, longer length of hospital stay (LOS) and even death (e.g., due to falls). Additionally, a decline in the ability to perform daily activities may increase the need for institutionalization in older persons (Fried et al. 2001). Several studies have investigated the possible association between anticholinergic drugs and delirium, LOS, physical function and mortality, but the results are inconsistent (Fox et al. 2014; Ruxton et al. 2015; Pasina et al. 2013; Lowry et al. 2012; Mangoni et al. 2016). This discrepancy might be caused by the methods used to assess anticholinergic drug use, which differ substantially between studies (Ruxton et al. 2015). In some studies, anticholinergic drug use is assessed with crude measures such as ‘exposed or not exposed’ and the total number of anticholinergic drugs taken by a person, whereas in other studies the specific anticholinergic load of the different drugs is taken into account. However, little is known about potential differences in results between these methods and whether the results can be compared. To the best of our knowledge no previous study has investigated the association between anticholinergic drugs and postdischarge institutionalization in acutely ill older patients.

The aim of the study was to investigate whether anticholinergic drug exposure on admission quantified according to three anticholinergic drug scales is associated with delirium on admission, LOS, postdischarge institutionalization and in‐hospital mortality in acutely ill older patients admitted to a geriatric ward.

## Materials and Methods

In this observational chart review study, we included acutely ill patients aged 65 and older who were admitted to the ward of geriatrics of the Erasmus University Medical Center, Rotterdam, The Netherlands, between 1 January 2012 and 31 December 2015. Patients were excluded if they were hospitalized for less than 3 days or if data regarding drug use or outcome measures were not available. Individual persons could be included more than once as patient in the study. The study was conducted according to the principles expressed in the Declaration of Helsinki.

### Demographic and clinical variables

All data were collected from the medical records and included age, sex, the estimated glomerular filtration rate (eGFR) on admission, drug use at the time of admission and the severity of comorbidities calculated with the Charlson Comorbidity Index (CCI) (Charlson et al. 1987). The CCI encompasses 19 medical conditions weighted with a score of 1–6, with total scores ranging from 0 to 37, with higher scores indicating a more severe burden of comorbidities. Data collected to determine outcome measures were: delirium status during the hospital stay, dates of admission and discharge, place of residence before and after discharge and in‐hospital mortality.

### Anticholinergic drug exposure

Dispensing records from the community pharmacy were preferentially used for recording all drugs in use by a patient at the time of admission. If this information was not available, we used correspondence letters of general practitioners or other referrers, or the medication history taken in the hospital. This information was additionally combined with patients’ self‐reports on over‐the‐counter drugs (reported in the medical record). When a drug was stopped 1 or more days prior to admission, we assessed whether there was a possibility that the drug was still present in a patient's body at the time of admission by calculating a time window of 5x the elimination half‐life of the drug.

Several anticholinergic drug scales have been developed previously that classify drugs according to their anticholinergic activity into four or five categories, ranging from no anticholinergic activity (score 0) to strong anticholinergic activity (score 3 or 4) (Salahudeen et al. 2015). Three of them, the Anticholinergic Risk Scale (ARS) (Rudolph et al. 2008), the updated version of the Anticholinergic Cognitive Burden (ACB) scale (Campbell et al. 2013) and the list of Chew et al. (2008) (hereafter called Chew), were used in the present study. Shortly, on the ARS, drugs are ranked based on their potential to cause central and peripheral anticholinergic adverse effects (score range: 0–3). Drugs assigned a score of 1 have a moderate anticholinergic potential and drugs with scores 2 and 3 have a strong and very strong potential, respectively. On the ACB, drugs are ranked based on their potential to have a negative effect on cognition (score range: 0–3). Drugs with a score of 1 are those with serum anticholinergic activity or in vitro affinity to muscarinic receptors, but without known clinically relevant cognitive effects. Drugs with established and clinically relevant cognitive anticholinergic effects were assigned a score of 2 or 3 (Boustani et al. 2008; Campbell et al. 2013). On the Chew, drugs are ranked based on in vitro serum anticholinergic activity measurements (score range: 0–3). Drugs with a score of 0.5 have an estimated anticholinergic activity of 0 at therapeutic doses, but may demonstrate some anticholinergic activity at higher doses. Drugs with a score of 1–3 demonstrate low to high anticholinergic activity across the therapeutic range (Chew et al. 2008).

In the present study, anticholinergic drug exposure on admission was defined as the use of drugs with anticholinergic properties, total number of anticholinergic drugs and total anticholinergic drug burden score (ADB), all quantified with the three anticholinergic drug scales. The ADB is the sum of scores assigned to each drug a patient is taking.

### Outcome measures

The outcomes of interest were delirium on admission, LOS, postdischarge institutionalization and in‐hospital mortality. On the ward of geriatrics, the diagnosis of delirium is made by geriatricians as part of daily clinical practice, according to the criteria of the Diagnostic and Statistical Manual of Mental Disorders, 4th and 5th edition (American Psychiatric Association 2000, 2013) and is based on daily psychiatric examination, medical and nursing notes, the Delirium Observation Screening scale scores, and information given by the patient's closest relative. In this study, reported diagnoses of delirium were extracted from the medical records. Delirium was defined as “present on admission” if the diagnosis was made within the first 2 days of the hospital stay. All other patients were considered as not having delirium on admission.

LOS was defined as the number of days a patient was hospitalized, with the first day of admission as day one. Patients who died during the hospital stay were not included in analyses of LOS.

Postdischarge institutionalization was defined as discharge to an institutional care facility rather than discharge to home. Patients who resided in an institutional care facility before admission and patients who died during the hospital stay were not included in analyses regarding postdischarge institutionalization.

In‐hospital mortality was recorded; all patients were included in the analyses.

### Statistical analyses

Differences in characteristics between patients with and without delirium on admission were compared using the Chi‐square test for categorical variables, the Mann–Whitney *U*‐test for non‐normally distributed continuous variables and the Student *t*‐test for normally distributed continuous variables.

Logistic regression analysis was performed to calculate odds ratios (OR) and corresponding 95% confidence intervals (CI) for delirium on admission, LOS, postdischarge institutionalization and in‐hospital mortality (dependent variables) according to different measures of anticholinergic drug exposure (exposure no/yes, total number of anticholinergic drugs and categories of ADB quantified with the ARS, the ACB and the Chew). LOS was divided into two groups based on the median value found in the overall sample (8.0 days). Number of anticholinergic drugs was treated as a continuous variable. ADB was divided into three categories: no ADB (for all scales score 0), moderate ADB (ARS and ACB score 1–2; Chew score 0.5–1.0) and high ADB (ARS and ACB score ≥ 3; Chew score ≥ 1.5); the first category was used as reference. All analyses were adjusted for age, sex, CCI and number of non‐anticholinergic drugs. Analyses of LOS, postdischarge institutionalization and in‐hospital mortality were additionally adjusted for delirium at any time during the hospital stay. Subsequently, analyses of LOS, postdischarge institutionalization and in‐hospital mortality were repeated in the group of patients with delirium on admission. Considering the suggested cholinergic deficiency in delirium (Hshieh et al. 2008) and the high prevalence of prolonged LOS, postdischarge institutionalization and in‐hospital mortality in patients with delirium (Witlox et al. 2010; Siddiqi et al. 2006), we hypothesized that the effect of anticholinergic drug exposure on aforementioned outcomes would be different in acutely ill older patients with delirium. LOS was divided into two groups based on the median value found in this group (10.0 days). Analyses were adjusted for age, sex, CCI and number of non‐anticholinergic drugs.

Repeated measures logistic regression models were fitted for all outcome measures using the Generalized Estimating Equations (GEE) method, to examine the effect of multiple inclusions per individual on the calculated estimates. Models were adjusted for the same covariates as the main analyses.

All statistical analyses were performed using Statistical Package for the Social Sciences (SPSS), version 21.0 (IBM Corp., Armonk, NY). Results were considered statistically significant at a *P* < 0.05.

## Results

**Figure 1:**
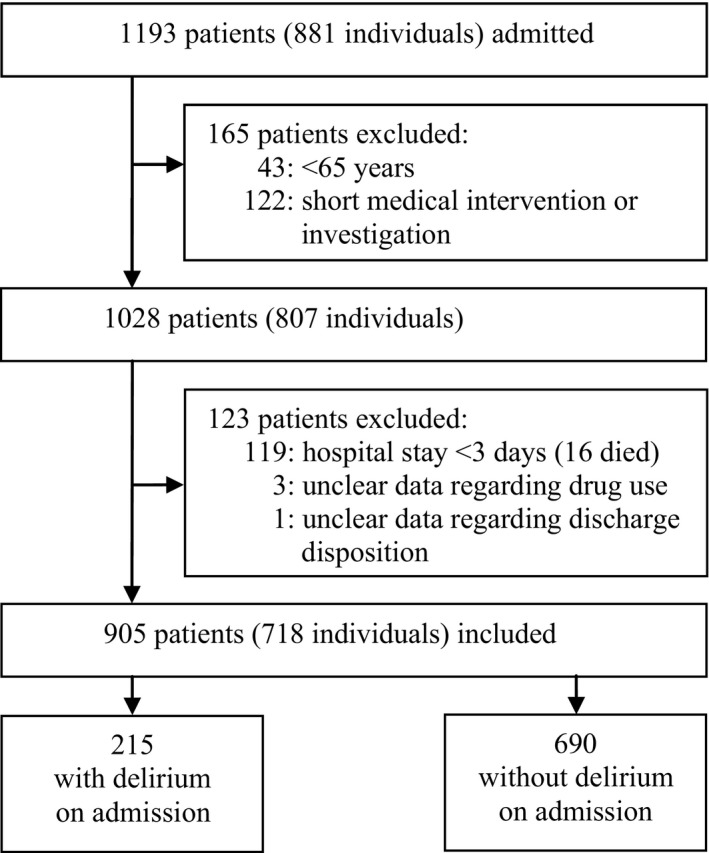
Flowchart of study sample selection.

A total of 1193 patients were admitted during the study period, of which 165 did not meet the inclusion criteria. Of the remaining 1028 patients, 123 were excluded: 119 were hospitalized for less than 3 days and four had unclear data regarding drug use or outcome measures. In total, 905 patients were included in the study; 215 of them (23.8%) had delirium on admission (Fig.**** 1). No statistically significant differences were found in sex distribution (men: 48.3% versus 41.5%, *P *=* *0.155) and mean age (81.0 ± 7.0 versus 81.0 ± 7.5, *P *=* *0.966) between patients who were included and those who were not. Baseline and discharge characteristics of the included patients are outlined in Table**** 1.

**Table 1:** Baseline and discharge characteristics of the overall study sample and stratified for delirium on admission

Characteristic	Overall sample *n* = 905	No delirium *n* = 690	Delirium *n* = 215	*P*‐value^a^
Male, *n* (%)	437 (48.3)	316 (45.8)	121 (56.3)	0.007^b^
Age, years, mean ± SD	81.0 ± 7.03	80.7 ± 7.1	81.9 ± 6.7	0.022^c^
Place of residence before admission, *n* (%)				0.035^b^
Home (with or without home care)	696 (76.9)	542 (78.6)	154 (71.6)	
Institutional care facility	209 (23.1)	148 (21.4)	61 (28.4)	
First time on the ward of geriatrics, *n* (%)	580 (64.1)	426 (61.7)	154 (71.6)	0.008^b^
CCI, median (IQR)	2.0 (1.0–4.0)	2.0 (1.0–4.0)	3.0 (1.0–4.0)	0.678^d^
eGFR, mL/min, median (IQR)	54.0 (35.0–75.0)	54.0 (35.0–75.0)	53.0 (36.0–74.0)	0.918^d^
Number of drugs, median (IQR)	8.0 (6.0–12.0)	8.0 (6.0–12.0)	8.0 (6.0–12.0)	0.963^d^
Use of at least one DAP, *n* (%)				
ARS	256 (28.3)	180 (26.1)	76 (35.3)	0.009^b^
ACB	644 (71.2)	488 (70.7)	156 (72.6)	0.605^b^
Chew	523 (57.8)	399 (57.8)	124 (57.7)	0.969^b^
Number of DAPs, median (IQR)				
ARS	0.0 (0.0–1.0)	0.0 (0.0–1.0)	0.0 (0.0–1.0)	0.011^d^
ACB	1.0 (0.0–2.0)	1.0 (0.0–2.0)	1.0 (0.0–2.0)	0.383^d^
Chew	1.0 (0.0–1.0)	1.0 (0.0–1.0)	1.0 (0.0–1.0)	0.585^d^
ADB score, median (IQR)				
ARS	0.0 (0.0–1.0)	0.0 (0.0–1.0)	0.0 (0.0–1.0)	0.015^d^
ACB	1.0 (0.0–3.0)	1.0 (0.0–3.0)	1.0 (0.0–3.0)	0.118^d^
Chew	0.5 (0.0–1.0)	0.5 (0.0–1.0)	0.5 (0.0–1.0)	0.474^d^
Delirium developed during the hospital stay, *n* (%)	45 (5.0)	45 (6.5)	n/a	n/a
Place of residence after discharge, *n* (%)				
Home (with or without home care)	448 (49.5)	389 (56.4)	59 (27.4)	<0.001^b^
Institutional care facility	392 (43.3)	260 (37.7)	132 (61.4)	
In‐hospital mortality, *n* (%)	65 (7.2)	41 (5.9)	24 (11.2)	0.010^b^
Length of stay, days, median (IQR)	8.0 (5.0–11.0)	7.0 (5.0–11.0)	10.0 (7.0–14.0)	<0.001^d^

SD, standard deviation; IQR, interquartile range; CCI, Charlson comorbidity index (range: 0–37); eGFR, estimated Glomerular Filtration Rate; DAP, drug with anticholinergic properties; ARS, Anticholinergic Risk Scale; ACB, Anticholinergic Cognitive Burden scale; ADB, Anticholinergic Drug Burden.

a No Delirium versus Delirium

b Chi‐square test.

c Student *t*‐test.

d Mann–Whitney *U*‐test.

### Delirium and length of stay

Table**** 2 presents the ORs and corresponding 95% CIs for delirium on admission and LOS ≥ 9 days according to different measures of anticholinergic drug exposure.

**Table 2:** Odds ratios for delirium on admission and prolonged length of hospital stay according to different measures of anticholinergic drug exposure

Variable	Delirium on admission		LOS ≥ 9 days	
	Delirium/no delirium	OR (95% CI)^a^	LOS ≥ 9/LOS < 9	OR (95% CI)^b^^,^ c
*ARS*				
Exposure to DAPs				
No	139/510	1.00 (ref)	274/330	1.00 (ref)
Yes	76/180	**1.73 (1.23–2.45)**	114/122	1.06 (0.77–1.48)
Number of DAPs				
Per drug	215/690	**1.38 (1.10–1.73)**	388/452	0.99 (0.79–1.24)
ADB score				
0	139/510	1.00 (ref)	274/330	1.00 (ref)
1–2	52/121	**1.70 (1.16–2.49)**	75/84	1.00 (0.69–1.45)
≥3	24/59	**1.83 (1.06–3.15)**	39/38	1.23 (0.73–2.07)
*ACB*				
Exposure to DAPs				
No	59/202	1.00 (ref)	113/133	1.00 (ref)
Yes	156/488	1.10 (0.77–1.59)	275/319	0.99 (0.71–1.38)
Number of DAPs				
Per drug	215/690	1.07 (0.94–1.23)	388/452	0.93 (0.82–1.06)
ADB score				
0	59/202	1.00 (ref)	113/133	1.00 (ref)
1–2	91/311	0.99 (0.67–1.46)	170/199	0.98 (0.70–1.39)
≥3	65/177	1.39 (0.89–2.18)	105/120	0.99 (0.66–1.51)
*Chew*				
Exposure to DAPs				
No	91/291	1.00 (ref)	161/193	1.00 (ref)
Yes	124/399	1.09 (0.78–1.51)	227/259	1.10 (0.81–1.49)
Number of DAPs				
Per drug	215/690	1.11 (0.94–1.31)	388/452	0.95 (0.82–1.12)
ADB score				
0	91/291	1.00 (ref)	161/193	1.00 (ref)
0.5–1	82/285	1.00 (0.71–1.43)	161/182	1.11 (0.81–1.54)
≥1.5	42/114	1.34 (0.85–2.11)	66/77	1.05 (0.69–1.62)

Values in bold are statistically significant (*P *<* *0.05).

OR, odds ratio; CI, confidence interval; LOS, length of hospital stay; DAPs, drugs with anticholinergic properties; ARS, Anticholinergic Risk Scale; ACB, Anticholinergic Cognitive Burden scale; ADB, Anticholinergic Drug Burden.

a Model adjusted for age, sex, Charlson comorbidity index and non‐anticholinergic drugs.

b Model adjusted for age, sex, Charlson comorbidity index, non‐anticholinergic drugs and delirium at any time during the hospital stay.

c Patients who died during the hospital stay were excluded.

After adjustment for age, sex, CCI and number of non‐anticholinergic drugs, we found that exposure to anticholinergic drugs according to the ARS was associated with an increased odds of having delirium on admission (OR = 1.73, 95% CI = 1.23–2.45). Each additional anticholinergic drug used by a patient was associated with a 38% increase in odds of having delirium on admission (OR = 1.38, 95% CI = 1.10–1.73). Both moderate and high ADB measured with the ARS were associated with an increased odds of having delirium on admission when compared to no ADB (OR = 1.70, 95% CI = 1.16–2.49 and OR = 1.83, 95% CI = 1.06–3.15, respectively). No associations were found between anticholinergic drug exposure quantified with the ACB and the Chew, and delirium.

After adjustment for age, sex, CCI, number of non‐anticholinergic drugs and delirium at any time during the hospital stay, no associations were found between anticholinergic drug exposure and LOS.

GEE logistic regression models for delirium on admission and LOS showed that the inclusion of individuals multiple times did not affect the estimates (data not shown).

### Postdischarge institutionalization and in‐hospital mortality

Table**** 3 presents the ORs and corresponding 95% CIs for postdischarge institutionalization and in‐hospital mortality according to different measures of anticholinergic drug exposure. After adjustment for age, sex, CCI, number of non‐anticholinergic drugs and delirium at any time during the hospital stay, we found that each additional anticholinergic drug used by a patient according to the ARS was associated with a 38% increase in odds of being institutionalized after discharge (OR = 1.38, 95% CI = 1.02–1.86). Additionally, a high ADB quantified with the ARS was associated with a 2.43 times higher odds of being institutionalized after discharge in comparison to no ADB (OR = 2.43, 95% CI = 1.24–4.75). No associations were found between anticholinergic drug exposure quantified with the ACB and the Chew, and postdischarge institutionalization.

**Table 3:** Odds ratios for postdischarge institutionalization and in‐hospital mortality according to different measures of anticholinergic drug exposure

Variable	Postdischarge institutionalization		In‐hospital mortality	
	Institutionalized/home	OR (95% CI)^a^^,^ b	Dead/alive	OR (95% CI)^a^
*ARS*				
Exposure to DAPs				
No	151/333	1.00 (ref)	45/604	1.00 (ref)
Yes	63/110	1.43 (0.95–2.14)	20/236	1.20 (0.67–2.15)
Number of DAPs				
Per drug	214/443	**1.38 (1.02–1.86)**	65/840	1.07 (0.73–1.58)
ADB score				
0	151/333	1.00 (ref)	45/604	1.00 (ref)
1–2	40/81	1.17 (0.74–1.85)	14/159	1.20 (0.63–2.29)
≥3	23/29	**2.43 (1.24–4.75)**	6/77	1.22 (0.47–3.13)
*ACB*				
Exposure to DAPs				
No	72/129	1.00 (ref)	15/246	1.00 (ref)
Yes	142/314	0.84 (0.56–1.25)	50/594	1.51 (0.80–2.84)
Number of DAPs				
Per drug	214/443	0.94 (0.80–1.11)	65/840	1.13 (0.90–1.41)
ADB score				
0	72/129	1.00 (ref)	15/246	1.00 (ref)
1–2	85/207	0.73 (0.48–1.12)	33/369	1.52 (0.79–2.93)
≥3	57/107	1.12 (0.68–1.86)	17/225	1.47 (0.66–3.25)
*Chew*				
Exposure to DAPs				
No	99/202	1.00 (ref)	28/354	1.00 (ref)
Yes	115/241	1.15 (0.80–1.67)	37/486	1.11 (0.64–1.91)
Number of DAPs				
Per drug	214/443	1.05 (0.85–1.29)	65/840	1.11 (0.84–1.46)
ADB score				
0	99/202	1.00 (ref)	28/354	1.00 (ref)
0.5–1	81/178	1.09 (0.74–1.62)	24/343	1.01 (0.56–1.83)
≥1.5	34/63	1.37 (0.80–2.36)	13/143	1.39 (0.66–2.92)

Values in bold are statistically significant (*P *<* *0.05).

OR, odds ratio; CI, confidence interval; DAPs, drugs with anticholinergic properties; ARS, anticholinergic risk scale; ACB, anticholinergic cognitive burden scale; ADB, anticholinergic drug burden.

a Model adjusted for age, sex, Charlson comorbidity index, non‐anticholinergic drugs and delirium at any time during the hospital stay.

b Patients who resided in an institutional care facility before admission and patients who died during the hospital stay were excluded.

After adjustment for age, sex, CCI, number of non‐anticholinergic drugs and delirium at any time during the hospital stay, no associations were found between anticholinergic drug exposure and in‐hospital mortality.

GEE logistic regression models for postdischarge institutionalization and in‐hospital mortality showed that the inclusion of individuals multiple times did not affect the estimates (data not shown).

### Analyses in patients with delirium on admission

Table**** 4 presents the ORs and corresponding 95% CIs for LOS ≥ 11 days, postdischarge institutionalization and in‐hospital mortality according to different measures of anticholinergic drug exposure in patients with delirium on admission. The association between anticholinergic drug exposure and postdischarge institutionalization found in the total group of acutely ill patients was not maintained in this subgroup. No associations were found between anticholinergic drug exposure and LOS and in‐hospital mortality.

**Table 4:** Odds ratios for prolonged length of hospital stay, postdischarge institutionalization and in‐hospital mortality according to different measures of anticholinergic drug exposure in patients with delirium on admission

Variable	LOS ≥ 11 days		Postdischarge institutionalization		In‐hospital mortality	
	LOS ≥ 11/LOS < 11	OR (95% CI)^a^^,^ b	Institutionalized/home	OR (95% CI)^a^^,^ c	Dead/alive	OR (95% CI)^a^
*ARS*						
Exposure to DAPs						
No	58/67	1.00 (ref)	55/43	1.00 (ref)	14/125	1.00 (ref)
Yes	30/36	1.07 (0.57–2.00)	28/15	1.57 (0.72–3.45)	10/66	2.08 (0.79–5.50)
Number of DAPs						
Per drug	88/103	1.00 (0.68–1.49)	83/58	1.36 (0.74–2.50)	24/191	1.17 (0.64–2.14)
ADB score						
0	58/67	1.00 (ref)	55/43	1.00 (ref)	14/125	1.00 (ref)
1‐2	20/24	1.02 (0.51–2.07)	18/11	1.40 (0.58–3.39)	8/44	2.52 (0.90–7.08)
≥3	10/12	1.18 (0.44–3.12)	10/4	2.08 (0.56–7.63)	2/22	1.15 (0.21–6.33)
*ACB*						
Exposure to DAPs						
No	26/29	1.00 (ref)	28/18	1.00 (ref)	4/55	1.00 (ref)
yes	62/74	0.99 (0.51–1.91)	55/40	0.85 (0.39–1.84)	20/136	2.19 (0.66–7.25)
Number of DAPs						
Per drug	88/103	0.79 (0.61–1.03)	83/58	0.75 (0.54–1.04)	24/191	1.22 (0.82–1.84)
ADB score						
0	26/29	1.00 (ref)	28/18	1.00 (ref)	4/55	1.00 (ref)
1‐2	38/41	1.05 (0.52–2.12)	32/26	0.75 (0.33–1.70)	12/79	1.94 (0.55–6.77)
≥3	24/33	0.88 (0.39–1.99)	23/14	1.12 (0.42–3.03)	8/57	2.99 (0.72–12.51)
*Chew*						
Exposure to DAPs						
No	39/43	1.00 (ref)	41/28	1.00 (ref)	9/82	1.00 (ref)
Yes	49/60	0.98 (0.53–1.79)	42/30	1.07 (0.52–2.19)	15/109	1.64 (0.62–4.33)
Number of DAPs						
Per drug	88/103	0.92 (0.67–1.26)	83/58	1.05 (0.72–1.55)	24/191	1.47 (0.92–2.35)
ADB score						
0	39/43	1.00 (ref)	41/28	1.00 (ref)	9/82	1.00 (ref)
0.5‐1	34/39	1.01 (0.52–1.95)	27/23	0.91 (0.42–1.98)	9/73	1.30 (0.45–3.77)
≥1.5	15/21	0.90 (0.39–2.08)	15/7	1.61 (0.55–4.72)	6/36	2.82 (0.80–9.95)

OR, odds ratio; CI, confidence interval; LOS, length of hospital stay; DAPs, drugs with anticholinergic properties; ARS, anticholinergic risk scale; ACB, anticholinergic cognitive burden scale; ADB, anticholinergic drug burden.

a Model adjusted for age, sex, Charlson comorbidity index and non‐anticholinergic drugs.

b Patients who died during the hospital stay were excluded.

c Patients who resided in an institutional care facility before admission and patients who died during the hospital stay were excluded.

GEE logistic regression models for the three outcome measures showed that the inclusion of individuals multiple times did not affect the estimates (data not shown).

## Discussion

In this study, we found that anticholinergic drug exposure, measured with the ARS, is associated with an increased prevalence of delirium and increased postdischarge institutionalization in acutely ill hospitalized older patients.

Our finding that anticholinergic drug exposure measured with the ARS is associated with delirium, is in agreement with the results of previous studies performed in critically ill patients (Wolters et al. 2015), palliative care patients (Zimmerman et al. 2014), patients with Parkinson's disease (Crispo et al. 2016) and older nursing home residents (Landi et al. 2014). Also, previous studies found no association between anticholinergic drug exposure, measured with the ACB or the Anticholinergic Drug Scale, and delirium in older hospitalized patients (Moorey et al. 2016; Campbell et al. 2011; Wolters et al. 2015). These findings strengthen the observation that results may differ depending on which scale is used when assessing anticholinergic drug exposure.

Several studies have investigated the possible relationship between anticholinergic drug exposure and LOS in older hospitalized persons (Pasina et al. 2013; Kidd et al. 2014; Lowry et al. 2011; Mangoni et al. 2016; Lowry et al. 2012). Three of them used the ARS and/or ACB and found, in line with our study, no association between anticholinergic drug exposure and LOS (Kidd et al. 2014; Pasina et al. 2013; Lowry et al. 2011). Mangoni et al. (2016) also used the ARS and found that anticholinergic drug exposure was only associated with prolonged LOS in older patients who were admitted during a non‐heat wave period. In contrast to our study, the previous study (Mangoni et al. 2016) included only older patients who were discharged home and did not exclude patients who were hospitalized for <3 days. Therefore, it might be speculated that the patients included in the study of Mangoni et al. (2016) were healthier and probably less frail than our study population. Lowry et al. (2012) also found that the use of anticholinergic drugs was associated with prolonged LOS in older hospitalized persons. However, they used an alternate anticholinergic drug scale and had previously found that the ARS was not associated with LOS in the same study sample (Lowry et al. 2011).

As far as we are aware this is the first study showing an association between anticholinergic drug exposure, measured with the ARS, and postdischarge institutionalization in acutely ill older patients. In a previous study, no association was found between anticholinergic drug use and nursing home admission within one year after hospital discharge (Narbey et al. 2013). However, Narbey et al. (2013) did not use an anticholinergic drug scale and treatment with anticholinergic drugs can have changed over time. Therefore, caution is warranted in extrapolating their results. A possible explanation for our finding that anticholinergic drug exposure, measured with the ARS, is associated with postdischarge institutionalization, might be an underlying decrease in functional performance. Several studies have found that anticholinergic drug exposure quantified with the ARS is associated with a reduced physical function in older persons (Pasina et al. 2013; Lowry et al. 2011; Landi et al. 2014; Lampela et al. 2013; Koshoedo et al. 2012).

Several studies have investigated the possible association between the use of anticholinergic drugs and in‐hospital mortality in older patients (Lowry et al. 2011, 2012; Mangoni et al. 2016; Kidd et al. 2014). Mangoni et al. (2016), Kidd et al. (2014) and Lowry et al. (2012) used the ARS, the ACB and the anticholinergic component of the Drug Burden Index respectively, and in line with our study, found no association. In a subgroup of older patients with hyponatremia, Lowry et al. (2011) reported that high ARS scores were associated with increased in‐hospital mortality.

To the best of our knowledge, no previous studies investigated the possible association between anticholinergic drug use and clinical outcomes in acutely ill hospitalized older patients with delirium. Recently, Kolanowski et al. (2015) found that the use of anticholinergic drugs according to the ACB was associated with prolonged LOS and reduced physical function, but not with discharge disposition in older persons with delirium who resided in a postacute care facility. In contrast to our study, their sample size was relatively small, all participants had dementia, were not acutely ill, and in the vast majority delirium was resolving.

Although no conclusions on causality can be drawn from this observational study, our results suggest that older persons who are exposed to anticholinergic drugs are at increased risk for delirium when they become acutely ill. Additionally, they might be at increased risk for postdischarge institutionalization independently of delirium. The question remains whether anticholinergic drugs ‘in general’ are associated with delirium and postdischarge institutionalization, since only the ARS was associated with them. Discrepancies in results between the ARS, ACB and Chew might be related to the large variation in number and ranking of drugs within each scale, which is caused by the different methods used to develop them. In all anticholinergic drug scales, the calculation of the ADB is based on the assumption that anticholinergic effects of different drugs are additive in a linear fashion. This might not be the case and therefore, inclusion of drugs without known clinically relevant anticholinergic effects might dilute possible associations. Therefore, it might be warranted to identify only drugs with established peripheral and cognitive anticholinergic effects in future studies. Furthermore, it might be possible that in delirium not only central anticholinergic effects may play a role, if any, but also peripheral anticholinergic effects. Blurred vision, urinary retention, constipation and confusion are risk factors for delirium and might explain why the ARS was associated with delirium. However, since we did not collect data on adverse effects, this remains speculative. In patients with delirium we found that the ARS was not associated with postdischarge institutionalization. It might be possible that the sample size was too small; other explanations could be that anticholinergic drugs play a minor role, if any, in the clinical course of delirium, or that anticholinergic drugs were stopped more frequently after admission since a cholinergic deficiency is still one of the most hypothesized causes of delirium (Hshieh et al. 2008).

### Limitations and strengths

This study has some limitations. First, the study design limits the ability to identify causal associations between the use of anticholinergic drugs and the outcome measures. Information on any changes in drug exposure during hospitalization was not collected and we cannot exclude that the treatment approach for the acute illness has influenced our results. Moreover, other health‐related factors, such as the reason for admission, the severity of illness, functional status and the degree of cognitive functioning can have influenced our results. In this study we were not able to score and adjust for the severity of illness and physical function, since information on those items was not always available. However, we have adjusted for the CCI in statistical models; therefore, we believe that we have provided an indirect adjustment for dementia. A comorbid cognitive disturbance, not diagnosed as dementia (yet), can still be a confounding factor. Second, the three anticholinergic drug scales were developed several years ago (the ARS and the Chew in 2008; the ACB was last updated in 2012) and do not include newer anticholinergic drugs. This might have led to an underestimation of the anticholinergic drug exposure, but we believe that our results are only minimally influenced by this. Third, the three anticholinergic drug scales do not take into account daily drug dose and treatment duration. Since it is likely that anticholinergic effects will be amplified with higher drug doses and longer treatment duration, this could have influenced our results. Fourth, our results are mainly based on information on prescribed drugs; minimal information was available on treatment adherence prior to hospitalization.

The study has several strengths. First, the findings were obtained in a relatively large sample size. Second, we used three anticholinergic drug scales within the same population which makes it possible to make clear comparisons between results. Third, the ARS, ACB and Chew provide a quick and simple measure of anticholinergic drug burden and are suitable for clinical practice.

## Conclusion

In this study, we found that anticholinergic drug exposure measured with the ARS, is associated with an increased prevalence of delirium on admission and increased postdischarge institutionalization in acutely ill hospitalized older patients.

Considering the fact that delirium and postdischarge institutionalization are associated with a very poor prognosis, future studies are needed to investigate whether regular medication reviews using the ARS are a useful tool in order to reduce complications and to preserve independent functioning in older persons.

## Author Contributions

Egberts, van der Craats, van Wijk, Alkilabe, van den Bemt, Mattace‐Raso: study concept and design. Egberts, van der Craats, van Wijk, Alkilabe: acquisition of data. Egberts, van der Craats, van Wijk, van den Bemt, Mattace‐Raso: analysis and interpretation of data. Egberts: preparation of the manuscript. Van der Craats, van Wijk, Alkilabe, van den Bemt, Mattace‐Raso: critical revision of the manuscript.

## Disclosure

All authors declare: no support from any organization for the submitted work; no financial relationships with any organizations that might have an interest in the submitted work in the previous 3 years; no other relationships or activities that could appear to have influenced the submitted work.

## Funding sources

None.

## References

[prp2310-bib-0001] American Psychiatric Association (2000). Diagnostic and Statistical Manual of Mental Disorders, 4th ed. Text revision. American Psychiatric Association, Washington, DC.

[prp2310-bib-0002] American Psychiatric Association (2013). Diagnostic and Statistical Manual of Mental Disorders, 5th ed. American Psychiatric Association, Arlington, VA.

[prp2310-bib-0003] Boustani M , Campbell N , Munger S , Maidment I , Fox C (2008). Impact of anticholinergics on the aging brain: a review and practical application. Aging Health 4: 311–320.

[prp2310-bib-0004] Campbell N , Perkins A , Hui S , Khan B , Boustani M (2011). Association between prescribing of anticholinergic medications and incident delirium: a cohort study. J Am Geriatr Soc 59(Suppl. 2): S277–S281. 22091573 10.1111/j.1532-5415.2011.03676.x PMC3234117

[prp2310-bib-0005] Campbell NL , Maidment I , Fox C , Kahn B , Boustani M (2013). The 2012 update to the Anticholinergic Cognitive Burden Scale. J Am Geriatr Soc 61(Suppl. 1): S142–S143.

[prp2310-bib-0006] Charlson ME , Pompei P , Ales KL , MacKenzie CR (1987). A new method of classifying prognostic comorbidity in longitudinal studies: development and validation. J Chronic Dis 40: 373–383. 3558716 10.1016/0021-9681(87)90171-8

[prp2310-bib-0007] Chew ML , Mulsant BH , Pollock BG , Lehman ME , Greenspan A , Mahmoud RA , et al. (2008). Anticholinergic activity of 107 medications commonly used by older adults. J Am Geriatr Soc 56: 1333–1341. 18510583 10.1111/j.1532-5415.2008.01737.x

[prp2310-bib-0008] Collamati A , Martone AM , Poscia A , Brandi V , Celi M , Marzetti E , et al. (2016). Anticholinergic drugs and negative outcomes in the older population: from biological plausibility to clinical evidence. Aging Clin Exp Res 28: 25–35. 25930085 10.1007/s40520-015-0359-7

[prp2310-bib-0009] Crispo JA , Willis AW , Thibault DP , Fortin Y , Hays HD , McNair DS , et al. (2016). Associations between Anticholinergic Burden and Adverse Health Outcomes in Parkinson Disease. PLoS ONE 11: e0150621. 26939130 10.1371/journal.pone.0150621 PMC4777375

[prp2310-bib-0010] Fox C , Smith T , Maidment I , Chan WY , Bua N , Myint PK , et al. (2014). Effect of medications with anti‐cholinergic properties on cognitive function, delirium, physical function and mortality: a systematic review. Age Ageing 43: 604–615. 25038833 10.1093/ageing/afu096

[prp2310-bib-0011] Fried TR , Bradley EH , Williams CS , Tinetti ME (2001). Functional disability and health care expenditures for older persons. Arch Intern Med 161: 2602–2607. 11718592 10.1001/archinte.161.21.2602

[prp2310-bib-0012] Hshieh TT , Fong TG , Marcantonio ER , Inouye SK (2008). Cholinergic deficiency hypothesis in delirium: a synthesis of current evidence. J Gerontol A Biol Sci Med Sci 63: 764–772. 18693233 10.1093/gerona/63.7.764 PMC2917793

[prp2310-bib-0013] Kidd AC , Musonda P , Soiza RL , Butchart C , Lunt CJ , Pai Y , et al. (2014). The relationship between total anticholinergic burden (ACB) and early in‐patient hospital mortality and length of stay in the oldest old aged 90 years and over admitted with an acute illness. Arch Gerontol Geriatr 59: 155–161. 24582945 10.1016/j.archger.2014.01.006

[prp2310-bib-0014] Kolanowski A , Mogle J , Fick DM , Campbell N , Hill N , Mulhall P , et al. (2015). Anticholinergic Exposure During Rehabilitation: cognitive and Physical Function Outcomes in Patients with Delirium Superimposed on Dementia. Am J Geriatr Psychiatry 23: 1250–1258. 26419732 10.1016/j.jagp.2015.07.004 PMC4691545

[prp2310-bib-0015] Koshoedo S , Soiza RL , Purkayastha R , Mangoni AA (2012). Anticholinergic drugs and functional outcomes in older patients undergoing orthopaedic rehabilitation. Am J Geriatr Pharmacother 10: 251–257. 22795433 10.1016/j.amjopharm.2012.06.003

[prp2310-bib-0016] Lampela P , Lavikainen P , Garcia‐Horsman JA , Bell JS , Huupponen R , Hartikainen S (2013). Anticholinergic drug use, serum anticholinergic activity, and adverse drug events among older people: a population‐based study. Drugs Aging 30: 321–330. 23475596 10.1007/s40266-013-0063-2

[prp2310-bib-0017] Landi F , Dell'Aquila G , Collamati A , Martone AM , Zuliani G , Gasperini B , et al. (2014). Anticholinergic drug use and negative outcomes among the frail elderly population living in a nursing home. J Am Med Dir Assoc 15: 825–829. 25282629 10.1016/j.jamda.2014.08.002

[prp2310-bib-0018] Lowry E , Woodman RJ , Soiza RL , Mangoni AA (2011). Associations between the anticholinergic risk scale score and physical function: potential implications for adverse outcomes in older hospitalized patients. J Am Med Dir Assoc 12: 565–572. 21514242 10.1016/j.jamda.2011.03.006

[prp2310-bib-0019] Lowry E , Woodman RJ , Soiza RL , Hilmer SN , Mangoni AA (2012). Drug burden index, physical function, and adverse outcomes in older hospitalized patients. J Clin Pharmacol 52: 1584–1591. 22167569 10.1177/0091270011421489

[prp2310-bib-0020] Mangoni AA , Jackson SH (2004). Age‐related changes in pharmacokinetics and pharmacodynamics: basic principles and practical applications. Br J Clin Pharmacol 57: 6–14. 14678335 10.1046/j.1365-2125.2003.02007.x PMC1884408

[prp2310-bib-0021] Mangoni AA , Kim S , Hakendorf P , Mayner L , Woodman RJ (2016). Heat waves, drugs with anticholinergic effects, and outcomes in older hospitalized adults. J Am Geriatr Soc 64: 1091–1096. 27160370 10.1111/jgs.14100

[prp2310-bib-0022] Moorey HC , Zaidman S , Jackson TA (2016). Delirium is not associated with anticholinergic burden or polypharmacy in older patients on admission to an acute hospital: an observational case control study. BMC Geriatr 16: 162. 27655289 10.1186/s12877-016-0336-9 PMC5031270

[prp2310-bib-0023] Narbey D , Jolly D , Mahmoudi R , Trenque T , Blanchard F , Novella JL , et al. (2013). Relationship between anticholinergic drug use and one‐year outcome among elderly people hospitalised in medical wards via emergency department: the SAFES cohort study. J Nutr Health Aging 17: 766–771. 24154649 10.1007/s12603-013-0349-4 PMC12878866

[prp2310-bib-0024] Pasina L , Djade CD , Lucca U , Nobili A , Tettamanti M , Franchi C , et al. (2013). Association of anticholinergic burden with cognitive and functional status in a cohort of hospitalized elderly: comparison of the anticholinergic cognitive burden scale and anticholinergic risk scale: results from the REPOSI study. Drugs Aging 30: 103–112. 23239364 10.1007/s40266-012-0044-x

[prp2310-bib-0025] Rudolph JL , Salow MJ , Angelini MC , McGlinchey RE (2008). The anticholinergic risk scale and anticholinergic adverse effects in older persons. Arch Intern Med 168: 508–513. 18332297 10.1001/archinternmed.2007.106

[prp2310-bib-0026] Ruxton K , Woodman RJ , Mangoni AA (2015). Drugs with anticholinergic effects and cognitive impairment, falls and all‐cause mortality in older adults: a systematic review and meta‐analysis. Br J Clin Pharmacol 80: 209–220. 25735839 10.1111/bcp.12617 PMC4541969

[prp2310-bib-0027] Salahudeen MS , Hilmer SN , Nishtala PS (2015). Comparison of anticholinergic risk scales and associations with adverse health outcomes in older people. J Am Geriatr Soc 63: 85–90. 25597560 10.1111/jgs.13206

[prp2310-bib-0028] Siddiqi N , House AO , Holmes JD (2006). Occurrence and outcome of delirium in medical in‐patients: a systematic literature review. Age Ageing 35: 350–364. 16648149 10.1093/ageing/afl005

[prp2310-bib-0029] Witlox J , Eurelings LS , de Jonghe JF , Kalisvaart KJ , Eikelenboom P , van Gool WA (2010). Delirium in elderly patients and the risk of postdischarge mortality, institutionalization, and dementia: a meta‐analysis. JAMA 304: 443–451. 20664045 10.1001/jama.2010.1013

[prp2310-bib-0030] Wolters AE , Zaal IJ , Veldhuijzen DS , Cremer OL , Devlin JW , van Dijk D , et al. (2015). Anticholinergic medication use and transition to delirium in critically ill patients: a prospective cohort study. Crit Care Med 43: 1846–1852. 26010688 10.1097/CCM.0000000000001094

[prp2310-bib-0031] Zeevi N , Pachter J , McCullough LD , Wolfson L , Kuchel GA (2010). The blood‐brain barrier: geriatric relevance of a critical brain‐body interface. J Am Geriatr Soc 58: 1749–1757. 20863334 10.1111/j.1532-5415.2010.03011.x PMC4667839

[prp2310-bib-0032] Zimmerman KM , Salow M , Skarf LM , Kostas T , Paquin A , Simone MJ , et al. (2014). Increasing anticholinergic burden and delirium in palliative care inpatients. Palliat Med 28: 335–341. 24534725 10.1177/0269216314522105

